# Rapid, label-free classification of tumor-reactive T cell killing with quantitative phase microscopy and machine learning

**DOI:** 10.1038/s41598-021-98567-8

**Published:** 2021-09-30

**Authors:** Diane N. H. Kim, Alexander A. Lim, Michael A. Teitell

**Affiliations:** 1 Department of Bioengineering, University of California, Los Angeles, CA 90095 USA; 2 Department of Pathology and Laboratory Medicine, University of California, Los Angeles, CA 90095 USA; 3 Molecular Biology Institute, University of California, Los Angeles, CA 90095 USA; 4 Eli and Edythe Broad Center for Regenerative Medicine and Stem Cell Research, University of California, Los Angeles, CA 90095 USA; 5 California NanoSystems Institute, University of California, Los Angeles, CA 90095 USA; 6 Department of Pediatrics, David Geffen School of Medicine, University of California, Los Angeles, CA 90095 USA; 7 Jonsson Comprehensive Cancer Center, David Geffen School of Medicine, University of California, Los Angeles, CA 90095 USA

**Keywords:** Biophysical methods, Imaging, Machine learning

## Abstract

Quantitative phase microscopy (QPM) enables studies of living biological systems without exogenous labels. To increase the utility of QPM, machine-learning methods have been adapted to extract additional information from the quantitative phase data. Previous QPM approaches focused on fluid flow systems or time-lapse images that provide high throughput data for cells at single time points, or of time-lapse images that require delayed post-experiment analyses, respectively. To date, QPM studies have not imaged specific cells over time with rapid, concurrent analyses during image acquisition. In order to study biological phenomena or cellular interactions over time, efficient time-dependent methods that automatically and rapidly identify events of interest are desirable. Here, we present an approach that combines QPM and machine learning to identify tumor-reactive T cell killing of adherent cancer cells rapidly, which could be used for identifying and isolating novel T cells and/or their T cell receptors for studies in cancer immunotherapy. We demonstrate the utility of this method by machine learning model training and validation studies using one melanoma-cognate T cell receptor model system, followed by high classification accuracy in identifying T cell killing in an additional, independent melanoma-cognate T cell receptor model system. This general approach could be useful for studying additional biological systems under label-free conditions over extended periods of examination.

## Introduction

Quantitative phase microscopy (QPM) is a label-free imaging technique with increasing adoption for cell characterization and identification studies^1^. With advances in machine learning, high-throughput QPM enabling the identification of cells of interest for isolation has high potential in basic research and for translation into clinical applications^2–4^. As previously demonstrated, combining QPM imaging with machine learning in fluid flow systems can increase the throughput of cellular characterization and the accuracy of classification between different cell types^5–7^. However, single time point measurements trade throughput for evaluations of the same cell or collection of cells over time, which impedes studies of time-dependent biological phenomena that require sequential measurements. Fluid-flow systems are also incompatible with studying adherent cells in their natural state because the cells need to be suspended to be processed on a fluidic platform. To address these shortcomings, there have been several attempts to incorporate time-dependence into QPM platforms with machine learning. For example, Vicar and colleagues reported a 75.4% prediction accuracy for classifying lytic versus apoptotic cell death in human prostate adenocarcinoma cells using time-dependent sequential quantitative phase data^8^.

A biological phenomenon of high interest is the cytotoxic activity of tumor-reactive T cells. The discovery of tumor antigen-recognizing T cells, their T cell receptors (TCRs), and cognate tumor-specific, cell surface antigens is an area of intense activity in cancer immunotherapy research. Adoptive cell transfer (ACT) and chimeric antigen receptor (CAR), especially CAR-T cell, cancer immunotherapies have shown high clinical potential, but are nonetheless limited by a range of patient responses and a paucity of dependable antigen-targeting TCRs that specifically attack tumor cells^9^. An exciting example of success in this approach is in hematologic malignancies, such as specific categories of leukemia or lymphoma, which frequently show high rates of response and tumor regression to immunotherapy approaches^8^. However, treatments of solid-tissue tumors with an adherent cell phenotype, such as melanoma, show patient response rate heterogeneity, with melanoma achieving one of the highest response rates at ~ 30%^10–12^.

Recent studies propose addressing burgeoning needs in time-dependent cell identification through multiple microfluidics and imaging approaches that utilize engineered, labeled non-adherent cellular systems^1,3^. The use of labeling allows high levels of cellular specificity, but presents a myriad of potential complications affecting biological integrity. These issues include but are not limited to the potential alteration of labeled cells through unintended molecular interactions, selection for another unintended trait in a subset of cells under study, and an extra manipulation step in translational use with clinical samples^13^. In particular, a method to screen for tumor-reactive T cells in naïve, fresh clinical samples would have broad importance for the development of new, targeted immunotherapies, especially in solid tumors where there is a shortage of highly specific and effective cancer immunotherapy targets^14^. QPM has shown potential for classifying white blood cells through flow-based methods^15–17^, however, currently there is not a time-dependent screening approach to identify T cell killing rapidly during image acquisition. Such an advance could span discovery stage studies in identifying new targets and TCRs for therapy, implementation approaches with streamlined processes for personalized therapy, and accessibility, with cost savings and time reductions in translational pipelines and clinical applications.

Previously, our lab quantified the label-free decrease of biomass in actively killed adherent tumor cells with a concurrent biomass increase in the activated T cells performing the killing over time using live cell interferometry (LCI)^2–4^. LCI is a label-free single-cell or cell-clump imaging application of QPM that is compatible with adherent and non-adherent cells^18^. LCI also enabled label-free studies of different cell fates in multiple biological contexts using threshold-based classification schemes based on QPM imaging features^19,20^. However, these prior studies were not easily translatable to clinical applications due to the extended time needed for post-imaging analysis and manual cell identification. To overcome these limitations and enable T cell killing classification, we provide a new rapid approach that is label-free and high-throughput using QPM with machine learning to monitor and identify T cell killing of target solid tumor cells. To demonstrate broad applicability of this approach in cancer immunotherapy, we used time-dependent input features from one adherent cancer cell line-cognate T cell-matched model system to train a machine-learning model that showed high classification accuracy in a second, independent adherent cancer cell line-cognate T cell-matched model system.

## Results

### Establishing a tumor cell and T cell co-culture test system

Our study goal was to create an automatic, rapid, label-free classification method using QPM data coupled to machine learning for accurate and reproducible identification of cells of interest. As a demonstration system, we chose to identify antigen-specific T cells that kill target melanoma cells. To begin, we generated a training dataset by monitoring and measuring changes in healthy growing tumor cells and tumor cells undergoing active killing using label-free QPM. For this purpose, we picked a well-characterized cytotoxic T lymphocyte (CTL) and target tumor-cell system used previously to study anti-cancer T cell reactivity^21^. This system consists of M202 human melanoma cells expressing surface Melanocytic Antigen Recognized by T lymphocytes (MART1, aka melan-A) and F5 TCR-transduced CD8+ T cells that kill human leukocyte antigen (HLA) matched MART1 + M202 cells^21,22^.

**Figure 1:**
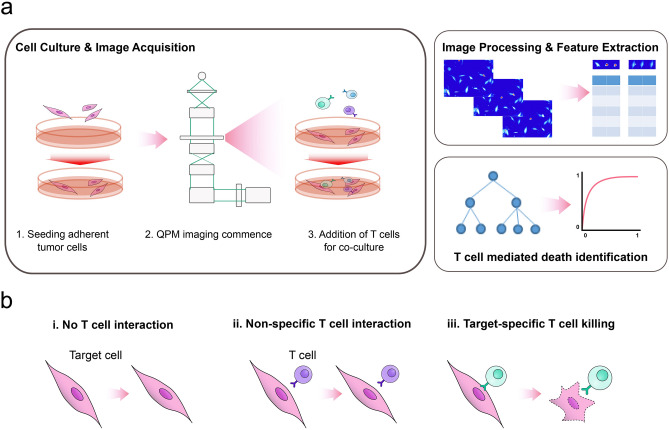
Schematic of the experimental test system and its analyses. (**a**) Schematic of co-culture system and QPM analysis. LCI imaging of M202 cells seeded into a culture dish establishes the unperturbed tumor cell biomass accumulation (growth) rate. Next, F5 TCR-transduced CD8+ T cells at a 2:1 ratio to M202 cells were added to the culture dish. Real time image analysis by image segmentation and software-enabled tumor cell tracking over time generates imaging features. These features are inputs for machine-learning models that attempt to accurately identify and classify T cell-mediated M202 melanoma cell killing. (**b**) M202 melanoma cells lacking recognition by T cells (i), or experiencing a non-specific T cell interaction (ii), continue to accumulate biomass and divide. By contrast, a HLA-restricted, F5 TCR-transduced CD8+ T cell and MART1 antigen-expressing M202 melanoma cell interaction activates tumor cell death identified by a machine-learning model classifier (iii).

M202 cells growing in culture were imaged using LCI before and after the addition of F5 TCR-transduced CD8+ T cells, with healthy melanoma cell growth prior to co-culture assessed by measurements of biomass accumulation over time (Fig. 1a and Supplementary Fig. S1). Following tumor-cell growth verification, the addition of F5 TCR-transduced CD8+ T cells at a 2:1 ratio to M202 cells established a CTL-tumor cell co-culture system (Fig. 1a)^23^. LCI images of the co-culture system were acquired every 15 m (“Methods”). This approach enabled the extraction of numerous raw QPM image-derived quantitative measurements, including optical, biophysical, and morphological features, which were collected and assessed over time from numerous individual target cells and CTLs (Table 1).

**Table 1:** Quantifiable features evaluated from QPM imaging data.

Feature	Description	Type
**Max intensity**	Maximum measured optical density per pixel in the given segmented area	Optical
Min intensity	Minimum measured optical density per pixel in the given segmented area	
**Mean phase shift**	Average optical density averaged over the given segmented area	
**Area**	Total segmented area of the cell	Biophysical
Biomass	Dry mass of the cell, summed over the given segmented area	
**Distance**	Displacement by a cell in between consecutive frames	
**Relative distance**	Distance divided by the area of the cell	
X-Coordinate	Coordinate along the X-axis of the frame of the center of the cell region	
Y-Coordinate	Coordinate along the Y-axis of the frame of the center of the cell region	
Convex area	Area of the smallest convex polygon that can contain the cell region	Morphological
**Eccentricity**	The ratio of the distance between the foci of the ellipse and its major axis length. The value is between 0 and 1	
**Equivalent diameter**	Diameter of a circle with the same area as the region. Computed as sqrt(4 × Area/pi)	
Extent	Ratio of region area to area of the total bounding box. Area/Area of the bounding box	
Filled area	Area of a rectangular box encasing the cell region	
**Major axis**	Length of the major axis of the ellipse encapsulating the cell region	
Minor axis	Length of the minor axis of the ellipse encapsulating the cell region	
Orientation	Angle between the x-axis and the major axis of the ellipse encircling the cell. The value is in degrees, ranging from − 90° to 90°	
**Perimeter**	Distance around the boundary of the region	
Perimeter 2	Perimeter with different edge weights in segmentation	
**Shape factor**	Area divided by its circumference or the length of its perimeter, P, (4πA/P)	
Solidity	Proportion of the area in the convex hull that are also in the region. Computed as Area/Convex Area	

Bolded are top ten parameters used for final evaluations.

### Identifying QPM features for classifying T cell killing events

QPM images from the co-culture system were organized into time tracks by connecting together consecutive measurement time points, as previously described^24^. Each time point corresponded to a specific imaging frame that contained information in the form of unique, quantifiable image features that cells exhibited at that specific time point. We only considered data from healthy growing tumor cells that showed growth by biomass accumulation before the co-culture system was established to exclude cell death not attributable to CTL activity.

**Figure 2:**
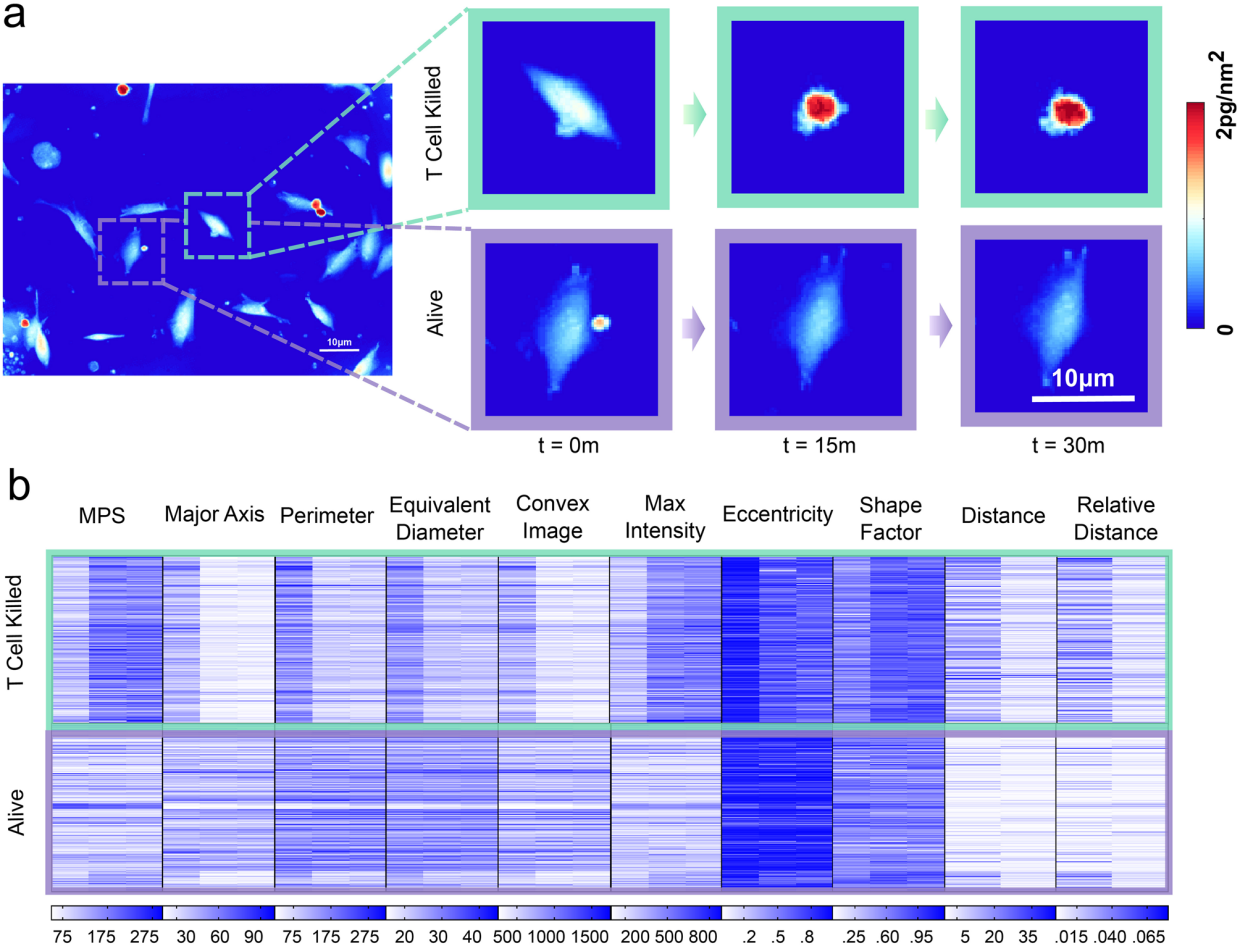
Progression of the quantitative phase density map during tumor-reactive T cell mediated killing and top ten extractable QPM features. (**a**) Representative LCI images of a single F5 TCR-transduced CD8+ T cell killing a MART1 + M202 melanoma cell over time. Phase density and mass distribution is shown in color scale ranging from 0 (background) to 2 pg/nm^2^. (**b**) Heat map of the top ten extracted QPM features of target cells for alive cell events versus T cell killed events. Each row represents an individual cell, and each major column represents a tumor cell feature. Each sub-column is a QPM image collection time point, here represented by 3 sub-columns for each imaging feature spanning 30 m. Tumor cell features in T cell killing events have more pronounced differences between imaging frames than alive tumor cell features, which are represented by changes in color intensity.

We manually annotated tumor cell imaging tracks to place M202 melanoma cells into two groups of classification, “alive” and “T cell killed”. In co-culture, melanoma cells had one of three observable fates. Fate 1 was growth over time without attack by proximal CTLs. Fate 2 was growth over time with a proximal CTL interaction, providing a potential failed tumor-cell killing event. Fate 3 was lysis and/or a prolonged biomass or cell area decrease verified by manual review during or after a physical interaction with a CTL (Figs. 1b, 2a)^21^. Imaging frames in which tumor cells were growing with or without T cell interactions (fates 1 and 2) as measured by biomass accumulation classified as alive, and frames in which tumor cells were dying specifically from T cell activity were classified as T cell killed (fate 3). A comparison of multiple quantitative imaging features (Table 1) over time between alive and T cell killed tumor cells revealed unique patterns. Tumor cells killed by CTLs showed feature changes that began with a reduction in projected cell area and increased cell density prior to measurable CTL-mediated lysis, determined through biomass loss, as established previously (Fig. 2b)^21^. To take advantage of differences between T cell killed and alive tumor cell features, we trained four independent machine-learning models to recognize morphological and biophysical changes that preceded CTL-mediated biomass loss from tumor cells.

**Figure 3:**
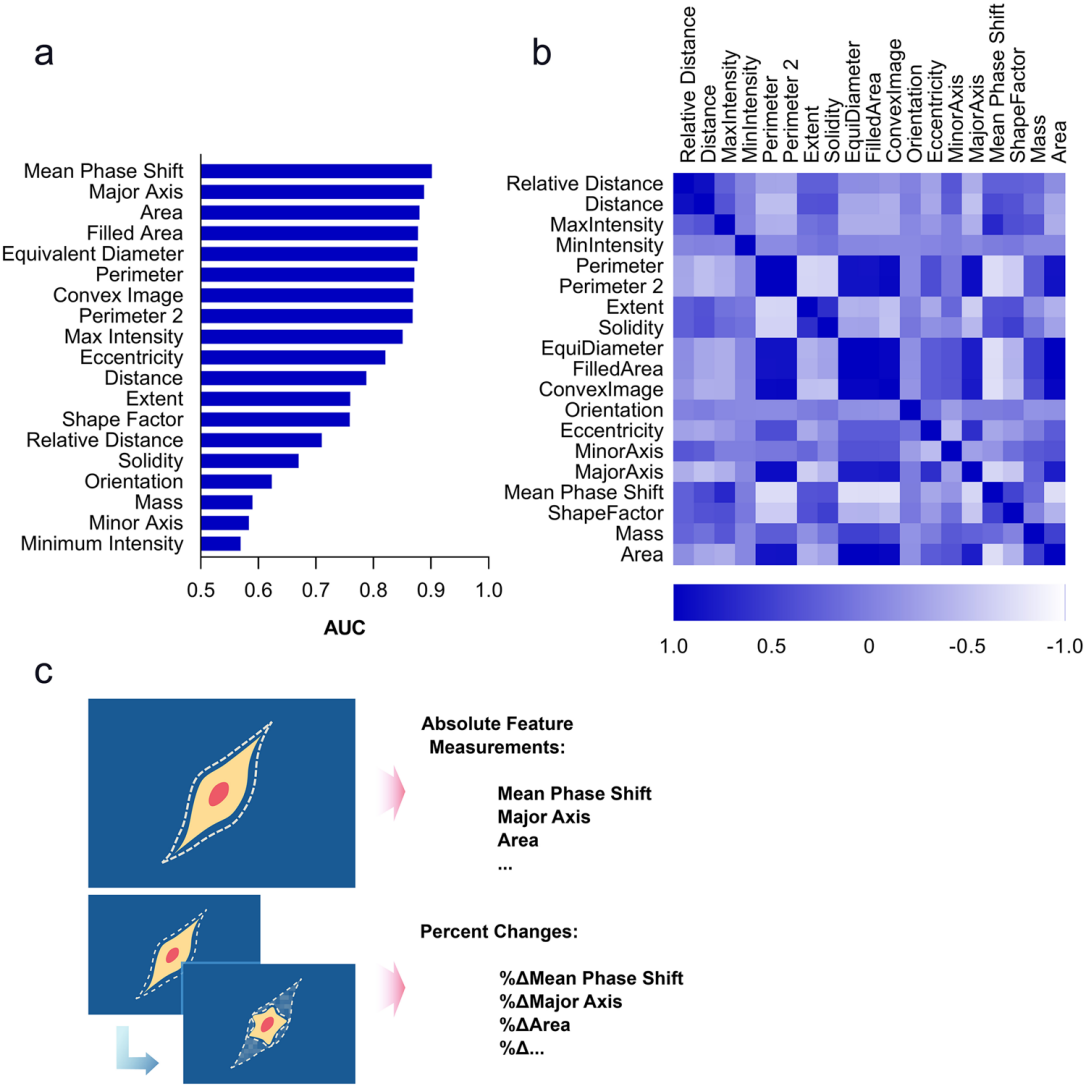
Univariate feature performance for classification. (**a**) Ranking of QPM features based on AUCs from univariate classification. (**b**) Heat map visual of a pairwise correlation matrix between 19 QPM imaging features analyzed by data extraction from quantitative images. Diagonal boxes represent autocorrelation of the feature with itself, with a value of 1. (**c**) Graphical representation of quantitative feature transformation. Absolute feature measurements are raw quantitative values of each feature collected from each LCI imaging frame. Percent changes were calculated from absolute feature measurements by dividing the difference of feature values from consecutive imaging frames by the feature value of the preceding frame.

We randomly placed collected QPM imaging features from individual M202 tumor cell tracks during co-culture with F5 TCR-transduced CTLs into training and validation datasets. To increase the accuracy of the examined machine learning models, we adjusted the number of T cell killed and alive cell events for equivalency in the training dataset^25^. In addition, to make model training as efficient as possible, we determined the most potent discriminating features for accurate classification of T cell killed compared to alive tumor cells by testing univariate feature performance (Fig. 3a). We evaluated four standard machine-learning classification models that include Bayes, Logistic Regression (LR), Support Vector Machine (SVM), and Random Forest (RF) for univariate classification power from the list of quantifiable QPM imaging features listed in Table 1. These features were then ranked according to their classification accuracy (Fig. 3a). Following feature ranking, the correlation or independence between each feature was calculated and presented in grid format (Fig. 3b). To reduce redundancies or less informative features in the imaging data landscape, and to increase the efficiency of model training, we eliminated the lower-ranked features with greater than 95% correlation with higher-ranked features (Supplementary Table S1) Using this approach, we curated QPM imaging features employed for model training to the top ten least overlapping input features (Table 1, bold font). Notably, the top ten performing features by univariate analysis for classification accuracy turned out to be the same for all four machine learning models under evaluation, even though the specific ranking order differed slightly between models.

### Transformation of raw QPM imaging features into machine learning model inputs

We next explored different feature input formats because raw data transformation can increase machine learning model classification accuracy. We converted raw imaging feature data into two different formats, absolute values (A) and percentage changes (P) from different numbers of tracking frames for each M202 target cell, including those that were T cell killed and those that remained alive^21,26^. Absolute value inputs were the raw quantitative values of the feature measurements. Percent change values were derived from the differences in the absolute values of feature measurements from successive imaging frames divided by the raw value of the preceding measurement (Fig. 3c). We tested five different input formats. Absolute 1 (A1) inputs were the absolute values of the top ten performing features of each tumor cell at a single time point. Absolute 2 (A2) inputs were features of each tumor cell at two consecutive time points. Absolute 3 (A3) inputs were features of each tumor cell from three consecutive time points. Percent 1 (P1) was the percent change in the top ten performing features of each tumor cell from two consecutive time points using values from A2 measurements. Percent 2 (P2) was the percent change in features of each tumor cell from three consecutive time points using values from A3 measurements (Fig. 3c). The transformation of raw absolute feature measurements into percent change had multiple potential benefits. First, this approach highlights the time-dependent change of tumor cell features^27^. Second, using percent changes removed scaling effects for each measured feature, with an example being that measured biomass ranges in the hundreds of picograms compared to, for example, eccentricity, with ranges from 0 to 1. Following the transformation of absolute values into percent changes, all the measured features range on a scale of 0–100%, removing the effects of different feature scales on the classification (“Methods”).

### Feature combinations efficiently identify tumor-reactive T cell killing events

**Figure 4:**
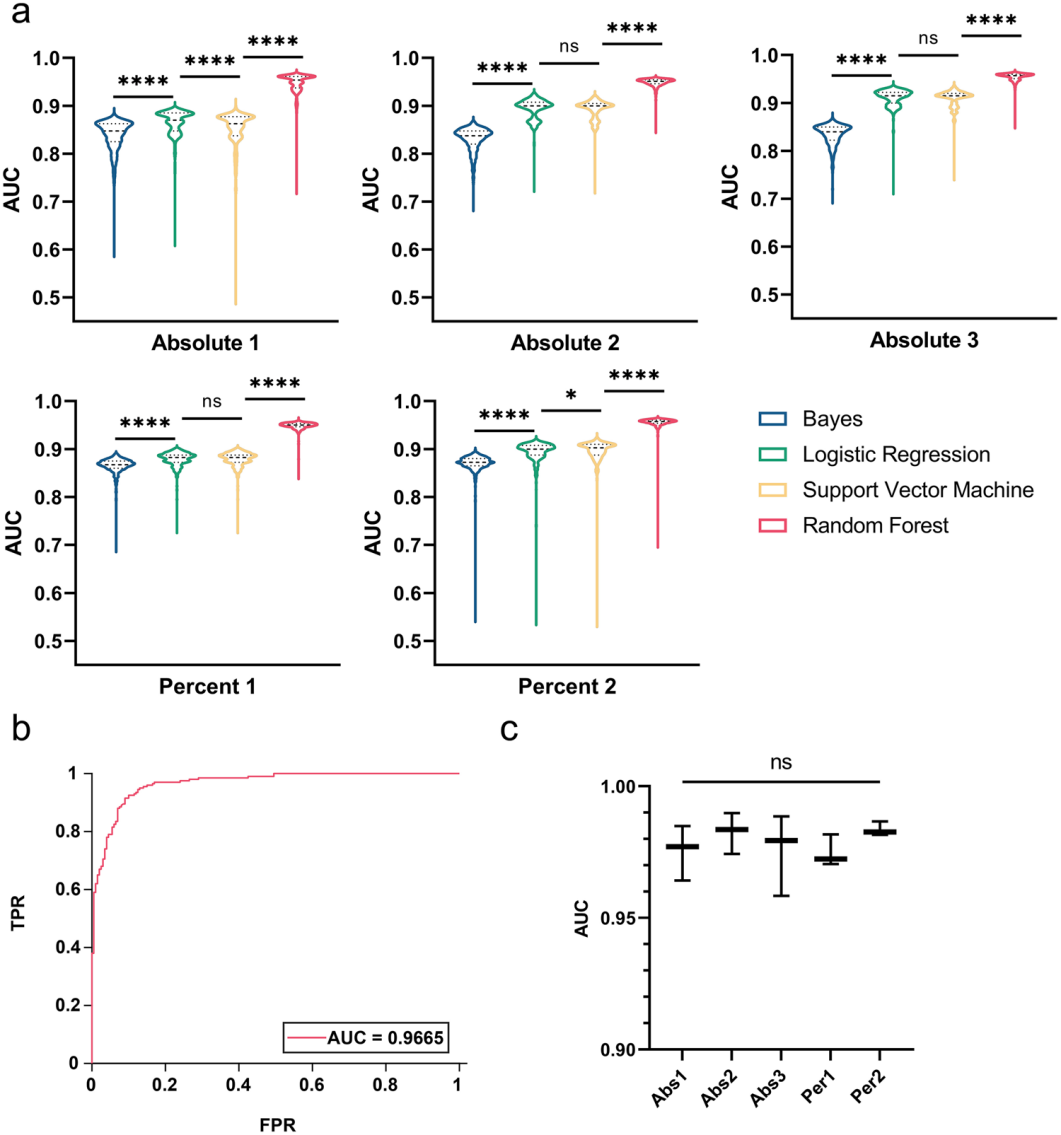
Training and validation performance of 1023 feature combinations using four different machine-learning models and input types. (**a**) Classification performance of models on training data. Each violin plot shows the performance of all 1023 feature combinations (n = 1023) used to train the model. Plots are not drawn to scale. ****denotes p < 0.0001. (**b**) ROC curve of the top performing feature combinations based on training data. The top classifier was a RF machine-learning model with input type P2, yielding an AUC of 0.9665. (**c**) Classification performance of top-performing RF models using the five data input types from three randomly populated QPM feature validation datasets. Percent 2 (Per2) showed the highest mean AUC and smallest standard deviation.

We tested the performance of different combinations of QPM measured features for rapidly identifying tumor-killing events using each of the four machine learning models. Using the top ten performing univariate features, we generated all combinations for each modeling input (e.g. A1, A2, A3, P1, and P2), which resulted in 1023 unique feature combinations per input type. We used these input combinations to train each machine-learning model with MATLAB. We plotted the classification results based on true positive rate and false positive rate into a receiver-operator characteristics (ROC) curve. The ‘area under the curve’ (AUC) of the ROC curve is a reliable metric for classification accuracy^5,28–30^ and was used to evaluate the training dataset classification accuracy of each trained machine-leaning model. To visualize the effects of specific features on classification performance, we compared AUC scores for the 1023 individual feature combination-trained classifications by input type, represented as violin plots (Fig. 4a). The highest performing classification accuracy occurred with RF for all five input types (p < 0.0001) (Fig. 4a). The top performing RF classification was trained using P2 input type, with an AUC of 0.9665 (Fig. 4b).

To validate our machine-learning model choice and to ensure that the trained model is not specialized only for the training data, we evaluated the RF classification model on the previously partitioned validation data set aside for F5-TCR transduced CD8+ T cell M202 melanoma cell killing. We further split this set aside data into three random datasets for assessment (each containing 67 live cell events and 67 T cell killing events). The performances of top input feature combinations for RF classification across different input types were statistically indifferent. P2 was chosen as the preferred input type because of its high mean AUC and smallest data spread using different feature combinations (Fig. 4c). P2 input features of the highest performing RF classifier included percent change in perimeter, major axis, maximum intensity, distance, and relative distance from three consecutive QPM imaging time points. Classification of the set aside validation data showed an average AUC of 0.97 for identifying F5 TCR-transduced CD8+ T cell killed M202 melanoma cells, providing confidence that the highly accurate classification performance was not specific for the training data.

### Trained model identifies T cell killing events in an independent system

**Figure 5:**
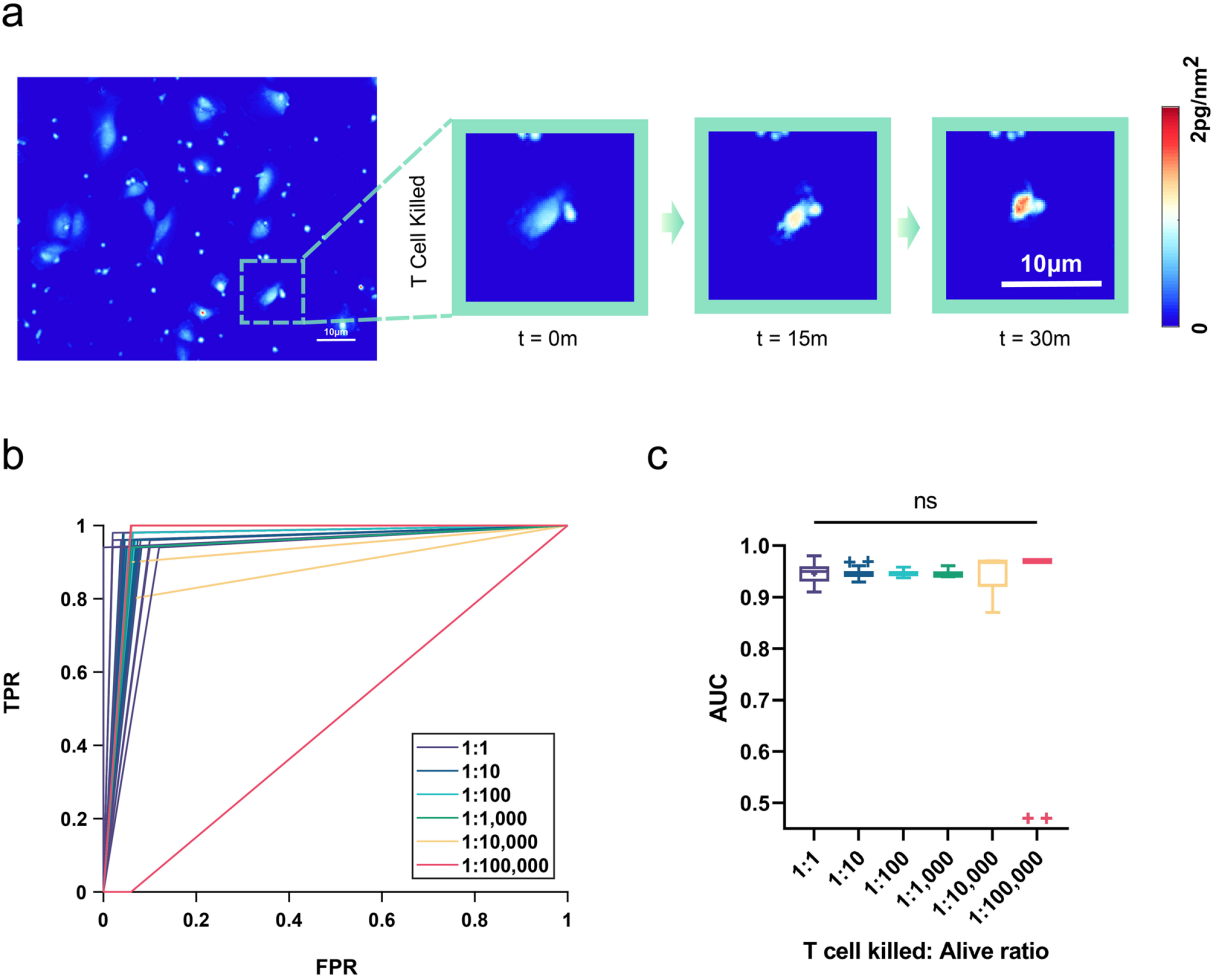
NY-ESO-1 TCR-transduced CD8+ T cell killing of M257-A2 melanoma tumor cells. (**a**) Representative images of a M257-A2 melanoma cell undergoing HLA-A2.1 restricted, anti-NY-ESO-1 antigen CTL mediated killing. (**b**) ROC curves using the RF model for the listed ratios of T cell killed to alive tumor cell classifications. (**c**) Classification performance by AUC of 30 randomly grouped datasets at the listed T cell killed to alive tumor cell ratios. We attribute outliers in 1:100,000 dilution group as having only one T cell killing event in the dataset, which was misclassified as alive.

A valuable machine learning classifier should perform well in multiple situations with similar input features without additional training. To evaluate the performance of the RF model with P2 type input features with no additional training, we picked a second, independent CTL-tumor cell co-culture demonstration system. This second system consists of slower growing M257-A2 melanoma cells engineered to express the cancer-testis antigen NY-ESO-1, and CD8+ T cells transduced with a cognate TCR that recognizes the NY-ESO-1 target tumor cell antigen. We used the same experimental set up, with growth established for M257-A2 tumor cells by measurements of biomass accumulation preceding the addition of CTLs recognizing the NY-ESO-1 tumor antigen, followed by mixed cell co-culture and LCI imaging over time (Fig. 5a). We processed the raw QPM imaging data from this second independent co-culture system in the same way as the F5 TCR-transduced CD8+ T cell, M202 melanoma co-culture system for classification.

With this second independent system, we simulated a clinically relevant biological sample that typically has a very low frequency of tumor-killing T cells by randomly assigning M257-A2 QPM imaging data into multiple datasets to represent a range of prevalence for tumor-killing T cells. The resultant in silico datasets ranged from one NY-ESO-1 TCR-transduced CD8+ T cell interaction (T cell killed) to one non-specific CD8+ T cell interaction (Alive), up to 1:100,000 T cell killed to Alive tumor cells, in multiple of 10 increments. We used the RF model with P2 inputs listed above trained solely on F5-TCR transduced CD8+ T cell, M202 melanoma data (Supplementary Table S2).

We observed accurate identification of T cell killed M257-A2 tumor cells over the range of CTL dilution, from 1:1 to 1:100,000, with AUC values consistently > 0.95 for NY-ESO-1 TCR-transduced CD8+ T cell killing of M257-A2 melanoma target tumor cells (Fig. 5b,c). This in silico exercise simulates accurate identification of T cell killing events in an independent mixed cell system without requiring the generation of new training data or retraining another machine learning classification model. It also demonstrates the potential to identify very rare tumor killing T cells within excised tumor sample materials using QPM and machine learning.

## Discussion

Clinical or translational applications in cell identification that incorporate machine learning would benefit from minimal perturbations or handling of biological samples, and from classification methods that are compatible with cell isolation and recovery. Classification schema should not require time- or material-intensive retraining for each independent, but stylistically related dataset, and should be applicable to a range of cell types. This is a special concern for the machine learning field, where a machine learning model trained on a specific training dataset may not work for different samples without retraining^31^. In the context of personalized cell therapy, cell identification and recovery should be fast to minimize turnaround time for patients in developing and delivering needed therapies.

One of the roadblocks for applications of machine learning in translational settings is the sheer size of good quality data required to train and validate a machine learning model. When exploring inputs for machine learning classifiers with biological applications, a common practice is to input as many features as possible to increase classification strength. Due to the relationship between statistical prediction power and the number of features in a training dataset, larger amounts of data are used to compensate for long lists of input features. However, for many clinical applications that involve low abundance or rare cells or events, a large number of data points may not be available for vigorous training. Therefore, important goals include (1) increasing the efficiency of training a classifier and (2) increasing the general applicability of a trained classifier for use with multiple different but stylistically related datasets.

Here, we used quantifiable features from serial QPM images as inputs for machine learning models and curated the number of features to reduce information redundancy. This approach achieved high prediction power from a relatively low number of training samples and identified a biological activity of interest, CTL killing of target tumor cells, without labels. Our classification approach including QPM features was also superior to running the same ML algorithms using two-dimensional-only features (Supplementary Fig. S2). To the best of our knowledge, this study is the first to show adherent tumor cell killing by tumor-specific T cells in label-free conditions over time, thereby expanding potential applications for QPM with machine learning. Our approach shows a high classification accuracy identifying T cell tumor killing in a mixed co-culture system (AUC > 95%)^8,31,32^. In this report, we demonstrated that the same classifier trained on just one T cell and cancer cell pair can achieve high classification accuracy in another, independent T cell and cancer cell pair. Looking to applications in additional T cell, cancer cell systems we suggest that optimization of our approach could require modulating data input combinations and temporal sampling cadence between measurements to account for differences in biological characteristics of the system. Such differences could include TCR affinity for target cancer cells, T cell and cancer cell types, the influence of other surface ligands or extracellular matrix, and others. Our proof of concept study that incorporates QPM, machine learning, label-free monitoring, and data analyses could enable future studies that combines automated cell picking to isolate and study T cells of interest, such as cloning tumor-reactive TCRs for additional opportunities in cancer immunotherapy.

## Methods

### Cells

M202 and M257-A2 HLA A2.1-restricted melanoma cell lines^33,34^, and cognate CD8+ T cell transduced anti-MART-1 and anti-NY-ESO-1 TCRs^21,34,35^ were obtained from the laboratory of Dr. Antoni Ribas (UCLA). We maintained melanoma cell lines in RPMI 1640 medium (ThermoFisher Scientific, Cat. #10040) supplemented with 10% Fetal Bovine Serum (FBS, Omega Scientific, FB-11), 0.7 mM non-essential amino acids (Gibco, Cat. #11140-050), and penicillin and streptomycin antibiotics (ThermoFisher Scientific, Cat. #15070063). T cells were maintained in C10 media (RPMI 1640 supplemented with 10% (v/v) FBS, 1% (v/v) Penicillin/Streptomycin, 10 mM HEPES, 50 μM β-mercaptoethanol, 1 × MEM NEAA, and 1 mM sodium pyruvate). with 10 µg/mL IL-2 (Peprotech, Cat #200). Cultured cells were routinely tested for mycoplasma with the Lonza Mycoalert Mycoplasma Detection Kit. Tumor cells were passaged at 85–90% confluency and maintained at 37 °C, 5% CO_2._

### Co-culture

Culture dishes (u-Dish 35 mm, low, u-Slide 4 well Ph+, ibidi) were coated with poly-l-lysine (Sigma-Aldrich, Cat. #P4832) for 1 h before seeding with M202 or M257-A2 melanoma cells at 1.8 × 10^4^ cells/ml. We transferred dishes with tumor cells to the LCI/QPM microscope stage, where they were imaged in the cell culture chamber to ensure healthy growth before T cells were added at a T cell to tumor cell ratio of 2:1. After 45 m passed to ensure that T cells settled into the plane of imaging, QPM data collection resumed for a total average period of 8–12 h.

### QPM image acquisition and processing

Microscopy was performed on a Zeiss Axio Observer A1 with stage-top incubation system (Zeiss). We performed quadriwave lateral shearing interferometry (QWLSI) quantitative phase imaging using a 20 × 0.4 NA objective. QPM data were captured with a SID4Bio QWLSI (Phasics) camera, which has an acquisition rate of 10 frames per second^36^. We acquired consecutive images at each location every 15 m for 8–12 h for 60 locations in the cell culture plate, with enough spacing between cells to enable successful image processing and segmentation. Custom MATLAB code was written to automatically process each new QPM image as the acquired image file was added to the directory. Feature analysis was performed using custom MATLAB scripts, as previously described^37^. Briefly, cells were segmented from the background using a combination of local thresholding and edge detection. Cell biomass was then computed from QPM data using an assumed cell average-specific refractive increment of 1.8 × 10^− 4^ m^3^/kg^19,38^. Individual cell information from the newest time point image were combined with those from two preceding time points and categorized into tracks based on a particle tracking code^21,25^. The track based on three time point frames was arranged into different input types (A1, A2, A3, P1, P2) and used as inputs for the classification. For the purpose of this study, we excluded non-single tumor cells in clusters of two or more, and tumor cells without biomass accumulation prior to co-culture with T cells, and their imaging tracks, from the analyzed datasets. Tumor cells showing biomass accumulation after commencement of co-culture with T cells were included as potential false positive T cell killed tumor cells. Each image processing and analyses event averaged < 2 m, starting from acquisition of the third time point image, through image analyses, to the end-result classification.

### Input type transformation

To generate percent change inputs (P1 and P2), the following formula was used:

$$\% \Delta_{n} { } = {\raise0.7ex\hbox{${(x_{n + 1} - x_{n} )}$} \!\mathord{\left/ {\vphantom {{(x_{n + 1} - x_{n} )} {x_{n} }}}\right.\kern-\nulldelimiterspace} \!\lower0.7ex\hbox{${x_{n} }$}},$$

where $\% \Delta$ denotes percent change, $x$ represents a quantitative (absolute) feature measurement, and subscripts $n$ and $n + 1$ represent consecutive imaging frames. Therefore, input P1 ($\% \Delta_{1}$) represents the percent change in raw feature values (A) calculated from the second and first imaging frames, such that:

$$P1{ } = {\raise0.7ex\hbox{${(A_{2} - A_{1} )}$} \!\mathord{\left/ {\vphantom {{(A_{2} - A_{1} )} {A_{2} }}}\right.\kern-\nulldelimiterspace} \!\lower0.7ex\hbox{${A_{2} }$}}.$$

Inputs that use data from more than one frame (i.e. A2, A3, P2) included the cumulative information from all the preceding frames. For example, A2 was comprised of absolute feature measurements from the second and first imaging frames, and P2 was comprised of percent changes calculated from third and second imaging frames, as well as P1, calculated from the second and first imaging frames.

### Manual dataset annotation

We classified tumor-specific T cell killing of M202 and M257-A2 melanoma cells in their respective co-culture experiments by manual review of LCI/QPM imaging frames. The requirements for classification as a T cell killed tumor cell were: (1) biomass accumulation prior to co-culture with T cells, (2) a visual interaction with a T cell, and (3) a decline in biomass and/or death by deformation or cell lysis. Tumor cells in co-culture that showed growth, including cell divisions, and non-killing interactions with T cells with continued biomass accumulation over time were classified as alive. This validated manual classification schema was previously reported^21^.

### Machine-learning model training

We used manually classified QPM data from F5 TCR-transduced CD8+ T cell, M202 melanoma cell co-cultures to train Bayes, LR, SVM, and RF machine-learning models with the MATLAB Statistics and Machine Learning Toolbox. From all QPM collected data, we randomly selected a subset for model training and set aside a subset for validation studies. For the training dataset, we used 200 QPM imaging tracks of alive, and 200 imaging tracks of T cell killed, tumor cells. Each imaging track contained information from at least three consecutive time points for each tumor cell. For validation studies, we further subdivided the randomly set aside QPM data into three randomly selected datasets of 67 alive and 67 T cell killed tumor cell tracks. AUC was calculated using MATLAB and plotted using Prism (Graphpad).

### In silico dilution

We generated a dilution series of NY-ESO-1 TCR-transduced CD8+ T cells to M257-A2 melanoma tumor cells from 61 T cell killed and 121,328 alive tumor cell tracks using custom MATLAB code to randomly mix and assign cells to 30 different datasets at the T cell to tumor cell ratios listed in Fig. 5b,c. 1:1 ratio datasets included 50 randomly selected T cell killed and 50 randomly selected alive cases. Similarly, 1:10 ratio datasets contained 50 randomly selected T cell killed and 500 randomly selected alive cases, and so on.

### Statistical analysis

Two-tailed unpaired student’s t-tests and one-way analysis of variance were used to test significance between different classification performance groups.

## Supplementary Information

Supplementary Information.
