# Supplementary material for: Rapid, label-free classification of tumor-reactive T cell killing with quantitative phase microscopy and machine learning
Source: Sci Rep. 2021 Sep 30;11:19448. doi: 10.1038/s41598-021-98567-8 (PMC8484462; doi:10.1038/s41598-021-98567-8)
Supplement: Supplementary file 1 — Supplementary Information. [file 41598_2021_98567_MOESM1_ESM.pdf]

Supplementary Information

Rapid, label-free classification of tumor-reactive T cell killing with quantitative phase microscopy and machine learning

Diane N.H. Kim, Alexander A. Lim, and Michael A. Teitell

Figure S1

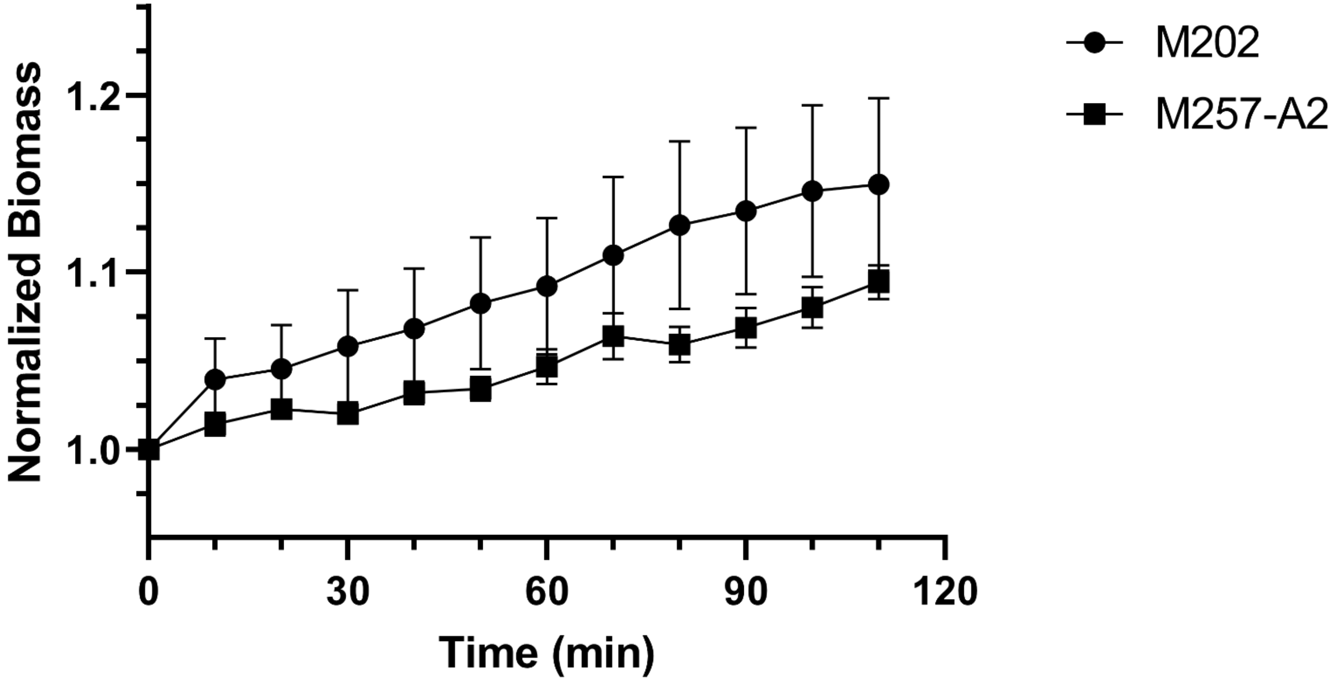

Supplementary Figure 1 Biomass accumulation measured by LCI during growth of melanoma cells before co-culture with TCR-transduced CTLs.

Figure S2

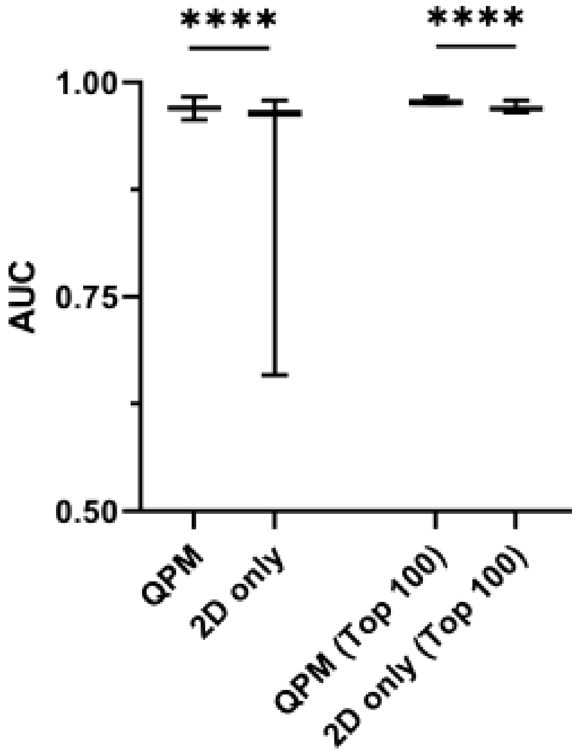

Supplementary Figure 2 A comparison of classification performance between identical ML feature combinations using QPM (QPM) versus two-dimensional (e.g. bright field-like) imaging features only (2D only). Feature combinations containing QPM features outperformed those with only two-dimensional features ($p<0.0001$). Top 100 performing feature combinations from each category were also compared ($p<0.0001$).

Table S1: Quantitative correlation plot for all QPM imaging features evaluated.

	Relative Distance	Distance	MaxIntensity	MinIntensity	Perimeter2	Perimeter	Extent	Solidity	EquiDiameter	FilledArea	ConvexImage	Orientation	Eccentricity	Minor Axis	Major Axis	Mean Phase Shift	Shape Factor	Mass	Area
Relative Distance	1.000	0.883	0.253	-0.044	-0.302	-0.302	0.255	0.252	-0.111	-0.111	-0.171	-0.027	-0.270	0.310	-0.365	0.247	0.242	0.203	-0.111
Distance	0.883	1.000	0.349	-0.015	-0.484	-0.484	0.356	0.365	-0.308	-0.305	-0.366	0.012	-0.314	0.256	-0.518	0.416	0.355	0.112	-0.305
MaxIntensity	0.253	0.349	1.000	-0.034	-0.378	-0.381	0.103	0.161	-0.363	-0.359	-0.360	-0.072	-0.186	0.004	-0.375	0.686	0.379	0.321	-0.359
MinIntensity	-0.044	-0.015	-0.034	1.000	-0.097	-0.094	0.058	0.105	-0.062	-0.061	-0.085	-0.087	-0.051	0.000	-0.078	0.020	0.058	-0.063	-0.061
Perimeter2	-0.302	-0.484	-0.378	-0.097	1.000	1.000	-0.671	-0.648	0.854	0.851	0.937	-0.095	0.389	0.065	0.902	-0.732	-0.595	0.275	0.851
Perimeter	-0.302	-0.484	-0.381	-0.094	1.000	1.000	-0.665	-0.647	0.855	0.852	0.938	-0.095	0.389	0.066	0.903	-0.733	-0.598	0.275	0.852
Extent	0.255	0.356	0.103	0.058	-0.671	-0.665	1.000	0.635	-0.399	-0.387	-0.508	0.008	-0.328	0.189	-0.610	0.347	0.359	-0.127	-0.387
Solidity	0.252	0.365	0.161	0.105	-0.648	-0.647	0.635	1.000	-0.286	-0.275	-0.526	0.072	-0.140	-0.052	-0.439	0.362	0.506	0.075	-0.275
EquiDiameter	-0.111	-0.308	-0.363	-0.062	0.854	0.855	-0.399	-0.286	1.000	0.997	0.955	-0.124	0.280	0.351	0.776	-0.743	-0.412	0.502	0.997
FilledArea	-0.111	-0.305	-0.359	-0.061	0.851	0.852	-0.387	-0.275	0.997	1.000	0.956	-0.134	0.281	0.349	0.774	-0.733	-0.399	0.499	1.000
ConvexImage	-0.171	-0.366	-0.360	-0.085	0.937	0.938	-0.508	-0.526	0.955	0.956	1.000	-0.164	0.286	0.336	0.802	-0.751	-0.484	0.397	0.956
Orientation	-0.027	0.012	-0.072	-0.087	-0.095	-0.095	0.008	0.072	-0.124	-0.134	-0.164	1.000	0.106	-0.233	0.013	0.017	0.055	-0.155	-0.134
Eccentricity	-0.270	-0.314	-0.186	-0.051	0.389	0.389	-0.328	-0.140	0.280	0.281	0.286	0.106	1.000	-0.470	0.632	-0.303	-0.203	0.019	0.281
Minor Axis	0.310	0.256	0.004	0.000	0.065	0.066	0.189	-0.052	0.351	0.349	0.336	-0.233	-0.470	1.000	-0.261	-0.168	0.130	0.302	0.349
Major Axis	-0.365	-0.518	-0.375	-0.078	0.902	0.903	-0.610	-0.439	0.776	0.774	0.802	0.013	0.632	-0.261	1.000	-0.677	-0.556	0.234	0.774
Mean Phase Shift	0.247	0.416	0.686	0.020	-0.732	-0.733	0.347	0.362	-0.743	-0.733	-0.751	0.017	-0.303	-0.168	-0.677	1.000	0.474	0.172	-0.733
Shape Factor	0.242	0.355	0.379	0.058	-0.595	-0.598	0.359	0.506	-0.412	-0.399	-0.484	0.055	-0.203	0.130	-0.556	0.474	1.000	-0.025	-0.399
Mass	0.203	0.112	0.321	-0.063	0.275	0.275	-0.127	0.075	0.502	0.499	0.397	-0.155	0.019	0.302	0.234	0.172	-0.025	1.000	0.499
Area	-0.111	-0.305	-0.359	-0.061	0.851	0.852	-0.387	-0.275	0.997	1.000	0.956	-0.134	0.281	0.349	0.774	-0.733	-0.399	0.499	1.000

*Green highlighted cells represent correlation values greater than 0.250, yellow highlighted cells represent correlation values greater than 0.500, red highlighted cells represent correlation values greater than 0.750. Diagonal rows represent correlation of a feature with itself, and therefore have values of 1.000.

Table S2: List of features and input type for best-performing classification model.

FEATURES OF TOP PERFORMING CLASSIFIER FOR M202-F5 TRAINING DATASET	INPUT TYPE
%∆Perimeter, %∆Major Axis, %∆Maximum Intensity, %∆Distance, %∆Relative Distance	P2
